# Biological Synthesis of Copper Nanoparticles Using Edible Plant *Allium monanthum*: Characterization of Antibacterial, Antioxidant, and Anti-Inflammatory Properties Using In Silico Molecular Docking Analysis

**DOI:** 10.3390/ma16206669

**Published:** 2023-10-12

**Authors:** Hyo Shim Han, Jeong Sung Jung, Young-Il Jeong, Ki Choon Choi

**Affiliations:** 1 Institute of General Education, Sunchon University, Suncheon 57922, Republic of Korea; kkruki@hanmail.net; 2 Grassland and Forages Division, National Institute of Animal Science, Rural Development Administration, Cheonan 31000, Republic of Korea; jjs3873@korea.kr; 3 Department of Dental Materials, College of Dentistry, Chosun University, Gwangju 61452, Republic of Korea

**Keywords:** copper nanoparticles, *A. monanthum*, electron microscope, anti-microbial, anti-inflammatory, in silico analysis

## Abstract

This study prepared copper nanoparticles using an edible leaf extract from *A. monanthum* (AM-CuNPs) via eco-friendly green synthesis techniques. The size, shape, crystalline nature and functional groups of the synthesized AM-CuNP particles were analyzed by a UV-VIS spectrophotometer and SEM, EDX, TEM, XRD and FT-IR instrumentation. The synthesized AM-CuNPs had spherical shapes with sizes in the range of 30–80 nm and were crystalline in nature. In addition, the AM-CuNPs were synthesized using various bioactive sources, including flavonoids, phenolic acids, alkaloids and sugars that were present in an aqueous broth of *A. monanthum*. Furthermore, the AM-CuNPs possessed good antibacterial properties against selected major disease-causing pathogenic bacteria, such as *E. coli*, *Salmonella typhi*, *Pseudomonas aeruginosa* and *Staphylococcus aureus*. The antioxidant activity of AM-CuNPs exhibited potent free radical scavenging activities in DPPH, ABTS and H_2_O_2_ radical assays. In addition, in silico analysis of the AM-CuNPs was performed, including ADME prediction, and molecular simulation docking on the secondary metabolites identified in the edible plant extract was used to evaluate their anti-inflammatory applications. In particular, the molecular docking scores showed that alliin, apigenin, isorhamnetin, luteolin and myricetin have sufficient binding energy and top values as inhibitors of the protein target involved in the inflammation signaling cascade.

## 1. Introduction

Metal nanoparticles (NPs) have been successfully synthesized from various sources, including plants, microorganisms and other sources of animal origins [1]. Recently, plant extracts have been used in numerous applications, such as in antimicrobial agents, photocatalytic substances, catalysis and for commercial uses [2]. To date, copper nanoparticles (CuNPs) have been synthesized from different plant extracts. For example, *Aloe vera*, *Magnolia kobus* and *Euphorbiaceae* sp. are used as reducing agents and capping molecules [3,4,5,6,7]. NPs are small, potent particles that contain a larger surface area on which biological entities such as DNA, probes, proteins, drugs and microorganisms can bind, adsorb and transport. NPs of different metals such as gold, silver, copper, zinc, platinum and iron are widely used in therapeutic and commercial applications. Recently, CuNPs have been spotlighted as critical components in future nanodevices and sensors due to their high catalytic activity, chemical stability, antimicrobial properties, electrical conductivity and biocompatibility [8,9]. CuNPs are promising nanomaterials that can be used in various biomedical and pharmaceutical applications, such as for antimicrobials, antivirals, wound healing, food packaging, water disinfection, surgical instrument manufacturing and pathogen detection assays [10]. CuNPs commonly have good antibacterial properties since they easily undergo oxidation and tend to dissolve in ionic aqueous media. CuNPs and ions can be considered as reductive species that can induce oxidative stress in pathogens through the generation of reactive oxygen species, which may damage cellular components, such as proteins, lipids and the nucleic acids of bacterial membranes, and suppress their multiplication and growth in the host [5,11].

The green-based synthesis of metal NPs has been extensively investigated, since their unique characteristics, such as shorter time and single-step reactions without the addition of any chemical reducing or stabilizing agents, facilitate the production of NPs with the desired sizes and shapes and high biocompatibility [12]. In addition, the formation of NPs during biosynthesis consists of three major stages: reduction of ions, clustering, and further NP growth. The secondary metabolites in plant extracts may play a critical role in the synthesis procedure. To date, different natural resources have been utilized for the green synthesis method: for example, plants, algae, bacteria, fungi, food waste, and so on. Plants and their active ingredients can provide a suitable medium for the large-scale production of various metallic NPs, and it becomes a novel and environmentally friendly process [7,8,9,10,11,12,13,14]. Plant-mediated NP synthesis has many advantages, such as ease of use, biological safety, the variety of active metabolites in the synthesis reaction, lower preparation time, and production of high-purity NPs. In addition, plants are a reservoir of different phytochemical compounds such as terpenoids, saponins, phenolics, flavonoids and sugars that act as capping, reducing and stabilizing agents for the biosynthesis of NPs [15].

*Allium monanthum* Max. (Dalrae: local Korean name) is a perennial herb belonging to the Liliaceae family. It is cultivated in the southeast region including Korea, China and Russia. Its bulbs and soft parts are edible and used in Korean traditional medicine [16,17]. The plant contains various ingredients, such as alliin, luteolin, myricetin, apigenin, isorhamnetin and sorbose. Particularly, isorhamnetin-3-O-β-D-glucoside is highly effective in inducing cell death in HT-29 human colon cancer cells and can activate cell apoptosis through condensation and reaction with the caspase protein cascade [18]. In addition, *A. monanthum* extracts are effective in reducing LDL lipid and total cholesterol levels in mouse liver samples. Therefore, *A. monanthum* extract contains functional substances that can aid the prevention and treatment of fatty liver disease in adults [19].

This study synthesized biological AM-CuNPs using *A. monanthum* aqueous extract and performed in vitro analysis to evaluate their properties, including morphologies, particle sizes and reducing capacity, which is involved in the redox function. Furthermore, antimicrobial activity against clinical pathogenic bacteria and the free radical scavenging properties of the AM-CuNPs were analyzed using DPPH, ABTS and hydroxyl radical assays. In silico anti-inflammatory mechanisms were also evaluated through computational modeling, simulation and analysis of the CuNPs.

## 2. Materials and Methods

### 2.1. Materials and Methods

All required reagents, including CuSO_4_·3H_2_O, ethanol, DMSO, hydrogen peroxide (H_2_O_2_), antibiotics and the ABTS assay kit, were purchased from Sigma-Aldrich Chem (St. Louis, MO, USA). The reagents were used at analytical grade without any further purification. The 2,2-diphenyl-1-picrylhydrazyl (DPPH) antioxidant assay kit was purchased from Abcam Co. Ltd. (Waltham, MA, USA).

### 2.2. Preparation of A. monanthum Plant Extract

The whole edible *A. monanthum* plants were purchased from the Pyeontek commercial food market, Korea, in 2022. The plant species were subjected to authentication by field experts and transported to the Grassland Forages Division, NIAS, Cheonan, Korea. The plants were cleaned using running tap water to remove soil particles and then allowed to air dry under shadow conditions for 12 h. Then, the plants were cut into small sizes, and extracts were prepared by adding 80 mL of sterile distilled water to 20 g of the plant samples. Afterwards, the mixtures were placed on a hot plate with a magnetic stirrer for 90 min at 120 rpm and then allowed to cool to room temperature. The solution was filtered through Whatman No.1 filter paper and stored at 4 °C.

### 2.3. Synthesis of Copper Nanoparticles Using A. monanthum Extract

NP biosynthesis was carried out as follows: An 80 mL copper sulphate solution (3 mM) was stirred slowly for 10 min. Next, 20 mL of aqueous *A. monanthum* extract was added to the mixture and stirred for 12 h at room temperature. During the reduction reaction, the color of the solution changed from pale blue to pale brown, which indicated the formation of AM-CuNPs. This was further confirmed by UV-Vis spectral analysis. Afterwards, the mixtures were centrifuged at 8000 rpm to harvest the solids, and the obtained pellet was dried in a hot oven at 100 °C and used for further analysis [20].

### 2.4. Characterization of the AM-CuNPs

The ultraviolet visible spectrum of the samples was scanned in the 250–650 nm range using a Shimadzu UV-2600 spectrophotometer (Shimadzu Corporation, Tokyo, Japan), and the optical density of the NP solution against the AM plant extract was recorded.

The nanostructure of the synthesized copper materials was analyzed by a scanning electron microscope (SEM, Carl Zeiss, Jena, Germany) with energy dispersive X-ray spectroscopy (XPS, ZEISS, Oxford Instruments, Abingdon, UK) to evaluate the elemental composition of the NPs.

A transmission electron microscope with high-resolution (HR-TEM, JEOL 2100, Peabody, MA, USA) was used to analyze the structure of the synthesized AM-CuNPs.

The Fourier transform infrared (FT-IR) spectra (FT-IR PerkinElmer, Yokohama, Japan) were recorded using the attenuated total reflection (ATR, Blagnac, France) method in the 4000–400 cm^−1^ range at room temperature.

An X-ray diffractometer (XRD-Shimadzu, PXRD-7000) equipped with CuKa (1.54060A) as the incident light was used to measure the crystalline nature of the AM-CuNPs.

### 2.5. Antibacterial Activity of AM-CuNPs

Biosynthesized AM-CuNPs were prepared in an aqueous medium (1 mg/mL) for antimicrobial assay by the agar well diffusion method. The antibacterial activity of AM-CuNPs was tested against four different bacterial pathogens including *E.coli, S. aureus, S. typhi* and *P. aeruginosa*. Briefly, Muller–Hinton agar plates were prepared, and a 100 uL suspension of each strain was spread evenly onto the surface of the plates. Then, an agar well was made in the plates and loaded with the CuNPs, control and 100 µL samples at different concentrations (25, 50, 75 and 100 µg). The sample-loaded agar plates were incubated at 37 °C for 48 h. Afterwards, the zone of inhibition was measured on the plates, and antibacterial activity was determined [21].

### 2.6. Antioxidant Activity of Synthesized AM-CuNPs

The antioxidant potential of the biosynthesized CuNPs was analyzed with three different free radical methods, including the 2,2-diphenyl-1-picrylhydrazyl (DPPH), ABTS and H_2_O_2_ scavenging assays. Experiments were performed based on the method previously reported by Rajeshkumar et al. [22].

### 2.7. In Silico Analysis of Anti-Inflammatory Effect of AM Synthesized CuNPs

#### 2.7.1. Ligand Preparation

The bioactive metabolites in *Allium monanthum* (Korean wild chive) were identified from the previously published literature [16,17]. The identified phytochemical structures were retrieved from the PubChem Application Programming Interface (API), in Simplified Molecular Input Line Entry System (SMILES) format and (3 Dimensional Standard Delay Format (3D-SDF). The chemical structures were downloaded from compound identification numbers (CIDs). In addition, all 3D-SDF files were minimized and converted to PDBQT (Protein Data Bank, Partial Charge (Q), & Atom Type (T)) format using Open Babel.

#### 2.7.2. ADME Pharmacokinetics Analysis

The SMILES file for each of the ligands was used for in silico absorption, distribution, metabolism and excretion (ADME) analysis using a SwissADME server (http://www.swissadme.ch), accessed on 29 August 2023, and performed with the normal parameter default settings. Predicted outer membrane penetration log kp using the in silico pharmacokinetics data was performed based on the Potts and Guy model calculation: log kp (cm/s) = 0.71 × log kow—0.0061 × MW—6.3, Here, MW is the molecular weight of the ligand compound and log kow is the octanol water partition coefficient, a physicochemical constant used to describe the lipophilic nature of the penetrant. Then, the compounds with high membrane transport capacity were selected for further analysis. Hierarchical clustering analysis was also conducted on a ChemMine web server (http://chemmine.ucr.edu/), accessed on 29 August 2023, as previously published by Fatoki et al. using the SMILES of the ligands [23].

#### 2.7.3. In Silico Target Prediction

The selected ligands that showed a high membrane permeability co-efficient based on the predicted pharmacokinetics analysis were used for target prediction with the SwissTarget Prediction server (http://www.swisstargetprediction.ch/), accessed on 30 August 2023, with homo sapiens selected as the target organism.

#### 2.7.4. Molecular Docking Studies

Molecular docking studies were performed as previously described by Fatoki et al. [24]. Briefly, the 3D structures of the three major molecular target proteins for anti-inflammation, namely FOXO, COX-2 and the NF-kB signaling cascade, were retrieved from the literature [25]. The structures of ligands that possessed high anti-inflammatory effects were exposed to 3D structure characterization using ChemSketch/ACDLab software, and files were saved in mol version format. PyMol software version 2.5 was used for ligand file conversion to PDB format from the mol version and also used for the preparation of target protein chain A with the removal of water molecules from the ligand sources. Both proteins and ligands were further prepared for docking using AutoDock Tools (ADT) v1.5.6 with the default GUI model settings, and the output source file was stored in PDBQT format. Furthermore, the observation of the in silico anti-inflammatory properties of the chosen bioactive compounds from *A. monanthum* was carried out using the molecular docking program AutoDock Vina v1.2.3 tools. After docking, close interactions between the targets binding with the ligands were analyzed and visualized using LigPlot+ on an ezCADD web server.

## 3. Results

### 3.1. Preliminary Confirmation of CuNP Synthesis Using UV-Vis Spectra

**Figure 1:**
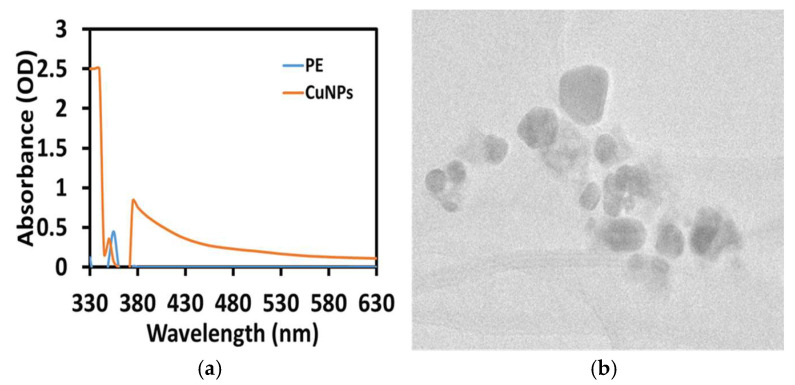
(**a**) UV-Vis spectra of CuNPs, (PE—*A. monanthum* plant extract alone); (**b**) TEM analysis of *A. monanthum* synthesized CuNPs.

This study successfully synthesized nanosized copper nanomaterials, which were preliminarily confirmed by exposing *A. monanthum* extract to room temperature and observing the color changes from pale blue to pale brown as evidence that CuNPs had been formed in the mixture due to the surface plasmon resonance. The study found that the surface plasmon resonance (SPR) peak position was 390 nm at 24 h and the second growth of nuclei final stable CuNPs occurred at an SPR peak of 350 nm (Figure 1a). The shifting peak of the SPR position due to the growth, clustering and nucleation of NPs was associated with the formation of NPs at different stages. Similarly, Mali et al. [26] (2020) suggested that the interaction between conduction electrons of metal NPs and incident photons was the key factor responsible for the color appearance and SPR characteristic peak obtained at 269 nm. Rajeshkumar et al. [22] reported that biosynthesized CuNPs using P. Americana exhibited gradual color changes from blue-green to brownish-black in the synthesis medium because of the SPR. The SPR absorbance was very sensitive when predicting the size and morphology of the NPs in the colloidal solution. We calculated the yield of synthesized AM-CuNP by initial weight of copper ions used for the synthesis and divided by the mass of final CuNP product obtained and by mass of the CuSO_4_. In this study, the final yield of the obtained AM-CuNPs was 65.48%.

### 3.2. Morphology and Size Characterization of AM-CuNPs by Electron Microscopy

Analysis using a transmission electron microscope clearly showed the size, shape and distribution of the AM-CuNPs. The size of the biosynthesized AM-CuNPs ranged from 20 to 80 nm. The size and shape distribution of the AM-CuNPs was highly uniform in the samples, and no aggregation was observed (Figure 1b). More uniform distributions and smaller particle sizes were obtained in the AM-CuNPs due to the reduction potential of the *A. monanthum* aqueous extract. Similarly, Amaliyah et al. [27] reported that some biomolecules in *P. retrofractum* extracts can significantly impact the synthesis medium and affect the size of the produced CuNPs.

**Figure 2:**
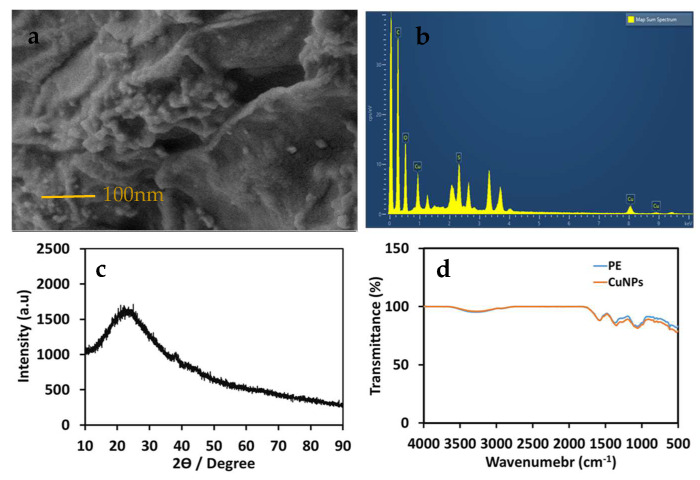
(**a**) FESEM images of biosynthesized AM−CuNPs; (**b**) EDX elemental composition of CuNPs; (**c**) Powder XRD pattern of AM-CuNPs; (**d**) FT−IR spectra.

The morphology and particle size of green synthesized AM-CuNPs were analyzed using FESEM-EDX techniques (Figure 2). The FESEM micrograph revealed that the synthesized CuNPs were spherical shapes with sizes of 20–80 nm, and some agglomerations were noticed due to the sampling preparation and sputter coating temperature. The size of the NPs was uniform, and the shapes were mostly spherical, even though a few triangular and rod shapes were observed. In addition, the FESEM images showed that the synthesized AM-CuNPs were of a uniform size or shape and tended to form a random aggregate. The EDX spectral analysis confirmed the composition of the synthesized AM-CuNPs (Figure 2b). The purity ratio of the synthesized CuNPs indicated 65% for Cu, and some weak signals for the elements C, O, Si, S and Ca were observed in the X-ray emission. These molecules originate from the macromolecules of plant derivatives such as flavonoids and phenolic compounds and can be used as reducing and capping agents for the synthesis of metal NPs. Similarly, Ghosh et al. [3] reported green synthesized JC-CuNPs, and in the elemental composition, the major ratio was Cu metal at 2–8 keV. In addition, other small peaks appeared for bioactive compound sources that were present in the plant extract that was used for the capping of the JC-CuNPs. In addition, the EDX analysis provided both qualitative and quantitative measurements for the elemental composition of the samples. Previously, Khashan et al. [28] found that Cu (71%) was the major element in CuNPs. Furthermore, O (15%) or C (13%) was present with other elements such as P, S, K and Tb, existing in the range of 1–2% [28]. Hence, these elements were present in NPs synthesized using the extract of P. retrofractum as a source.

### 3.3. XRD Analysis of Biosynthesized AM-CuNPs

Figure 2c shows the X-ray diffraction spectrum of biosynthesized AM-CuNPs. The XRD spectrum revealed intense diffraction peak 2θ values of 43.55°, 51.30° and 72.45° corresponding to the planes of (111), (200) and (220), respectively, which represent the bcc of copper metals. In addition, the peak at 29.85° showed that impurities were present due to the plant metabolites in the CuNPs. The XRD peaks were assigned in comparison with the standard powder diffraction spectrum of the Joint Committee on Powder Diffraction Standards (JCPDS card no. 89-2838) [26]. Hence, the peak positions were consistent with the metallic copper of a crystalline nature reported earlier. Recently, Narayanan et al. reported strong peaks at 2θ = 32.1°, 35.2°, 39.5°, 49.5°, 53.2°, 62.3°, 67.2°, 68.5° and 75.2°, corresponding to the (100), (002), (111), (202), (020), (113), (311), (220) and (222) planes, respectively, which are a perfect match with copper NPs [29]. In addition, the sharp intense peaks strongly supported the formation of copper NPs with an extremely crystalline nature, having values similar to the fcc cubic phases.

### 3.4. FT-IR Spectra of Biosynthesized AM-CuNPs

FT-IR analysis could provide information regarding the functional groups of the phytochemicals that participated in the synthesis of the AM-CuNPs and their stabilization. Figure 2d shows the FT-IR spectra of biosynthesized AM-CuNPs and the *A. monanthum* extract. The FT-IR spectrum results revealed broadband peaks in the 3400–2400 cm^−1^ range, indicating the presence of (O-H) assigned carboxylic acids, alcohols and phenols in the samples. In addition, the peak at 1574 cm^−1^ was assigned to the C=C alkenes and aromatic compounds. A peak at 1372 cm^−1^ was assigned to the C-O bond of esters, alcohols and carboxylic acids, and the presence of aromatic compounds was noticed by two strong peaks at 823 cm^−1^ and 780 cm^−1^, which indicated C-H aromatic compounds in both the AM-CuNPs and AM extract samples. The smaller peaks at 616 and 524 cm^−1^ were assigned to the aromatic bending vibration of the CH-alkane groups. Therefore, these functional groups indicated the presence of possible reductive substances on the AM-CuNPs and involvement of the reduction reaction from the *A. monanthum* extract. Similarly, Mali et al. [26] reported that biosynthesized CuNPs show distinctive characteristic bands at 3264.52 and 1636.62 cm^−1^, corresponding to the –NH_2_ assigned to primary amine and the OH stretching corresponding to the carboxylic acids, respectively, which are present in the leaf extract of *C. paniculatus* [26]. Thus, the results illustrate that the biosynthesized CuNP surface contains various functional groups that are involved in the reduction and capping of metal NPs that can enhance their biological applications. Furthermore, Sadia et al. [30] reported that biosynthesized CuNPs using *S. didymobotrya* root extract have new chemical linkages, which include the hydroxyl and carbonyl groups of amino acids, on their surface [30]. These bioactive substances play a role in the biosynthesis of NPs, such as inducing the reduction, stabilizing the metal ions and eventually the potent antibacterial and antioxidant functions. In addition, Vijayakumar et al. [31] suggested that the methanolic extract of Myristica fragrans seeds contains various functional group entities including the carbonyl, alkane, ether and amine groups of biological compounds such as tannins, phenolics, flavonoids, steroids and glycosides that activate the synthesis of metal NPs and enhance the functional mechanisms against pathogens without any adverse effect for humans and the environment [31].

### 3.5. Assessment of Antibacterial Activity of AM-CuNPs

**Figure 3:**
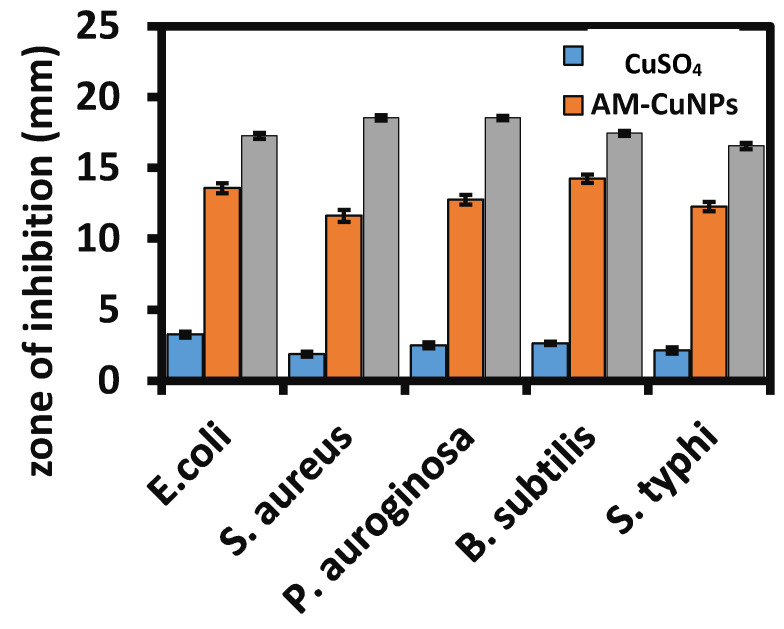
Antibacterial analysis of AM-CuNPs against pathogenic bacteria.

The copper metals have proven excellent antibacterial activity against some pathogenic bacterial strains. This study evaluated AM-CuNP nanosized materials against various Gram-positive and Gram-negative pathogenic bacteria. Figure 3 and Table 1 show the antibacterial activity of *A. monanthum* mediated CuNPs against clinically pathogenic bacteria. The maximum zone of inhibition was measured in the Gram-positive bacteria Streptococcus aureus, with a zone diameter of 18.65 ± 0.15 mm at a concentration of 100 uL of AM-CuNPs. The least activity was observed in *E. coli*, with a zone diameter of 11.25 ± 0.25 mm at 100 µL. In addition, the AM-CuNPs showed moderate activity against *S. typhi* and *P. aeruginosa*, with a zone diameter of 12.55 ± 0.35 and 13.65 ± 0.42 mm, respectively. Hence, the results demonstrated that biosynthesized AM-CuNPs are more active against Gram-positive bacteria than Gram-negative bacteria, which is possibly due to the difference in the cell components and structure of the cell membrane of pathogens. In addition, the antibacterial activity of bio-NPs could be due to the presence of bioactive metabolites on the surface of NPs acting as capping and stabilizing agents. Accordingly, CuNPs have promising antibacterial activity against *S. aureus* compared to the standard antibiotic such as ampicillin [32]. Recently, Ramasubbu et al. [33] reported that the antibacterial activity of Sg-CuO NPs was potent against *E. coli*, *P. aeruginosa* and *S. aureus* when compared with streptomycin (10 mg/mL) [33]. Furthermore, the concentration of the NPs increased as pathogen growth decreased, due to the dose-dependent responses of Sg-CuO against the pathogenic organisms. The Sg-CuO NPs strongly inhibited the growth of *E. coli* at 125 µg/mL and showed the highest zone of inhibition of 14 ± 1.39 mm in diameter. The results were compared to streptomycin antibiotics (17 ± 0.46 mm). Similarly, the CuNPs synthesized with star anise (Illicium verum) and nutmeg (Myristica fragrans) exhibited good antibacterial activity against *S. aureus*, with an inhibition zone of 1.33 ± 0.089 cm and 1.06 ± 0.073 cm, respectively [31].

### 3.6. Antioxidant Activity of AM-CuNPs

**Figure 4:**
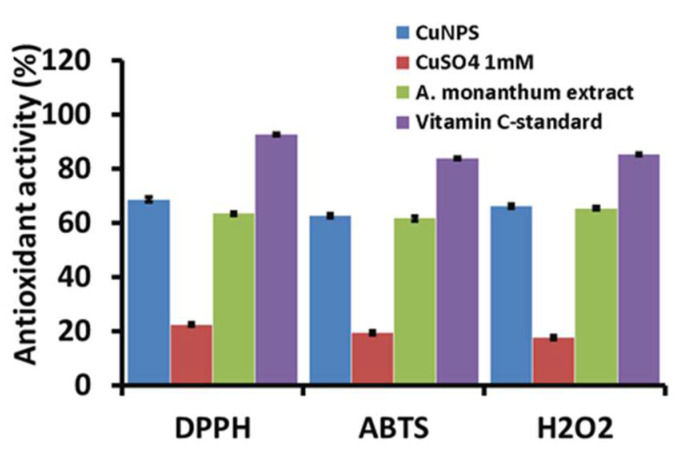
Antioxidant analysis of AM-CuNPs using DPPH, ABTS and H_2_O_2_ radicals.

The antioxidant behavior of *A. monanthum* biosynthesized CuNPs was tested by different free radical scavenging assays using DPPH, ABTS and H_2_O_2_ scavenging in vitro methods. The DPPH, ABTS and H_2_O_2_ free radical scavenging activity of AM-CuNPs is shown in Figure 4 and Table 2. DPPH has been studied widely due to its stable free radical presence and to evaluate the reducing potential of various natural and commercial substances. The study found that the *A. monanthum* synthesized AM-CuNPs have moderate scavenging activity (68.48 ± 0.85% at 100 µg/mL) against DPPH free radicals. The DPPH antioxidant behavior of the AM-CuNPs may be due to the presence of phenolic compounds, flavonoids, terpenoids and sugars in the plant extract used for the NP production. In addition, the DPPH free radical scavenging potential of the AM-CuNPs is very close to the ascorbic acid standard at the same dose. The higher concentration of AM-CuNPs has a significant effect on the DPPH radical scavenging activity compared to lower concentrations. Thus, the AM-CuNPs have dose-dependent activity [10].

The ABTS assay is a colorimetric method that strongly colors the cation radicals of ABTS•^+^, which accepts hydrogen atoms or electrons supplied by antioxidant substances. Figure 4 shows the ABTS cation scavenging activity of AM-CuNPs, AM-plant extract and the ascorbic acid standards. The maximum ABTS+ cation scavenging activity was shown by the AM-CuNPs (72.55% ± 0.65), and the ascorbic acid standard (79.55% ± 0.55) was slightly higher than the copper nanoparticles at 100 µg/mL concentrations. However, the synthetic drug has some adverse effects, which is a limitation of commercial products. Therefore, the AM-CuNPs could be potent sources for the ABTS+ cation scavenging activity. Similarly, Apak et al. [34] measured the antioxidant capacity of phenolic compounds (Ph-OH) via an ABTS assay and found that the test compounds decreased the ABTS•^+^ color by intercepting the initial oxidation, thereby directly preventing the ABTS•^+^ radical cation formation in the solution.

This study also evaluated the H_2_O_2_ scavenging effects of AM-CuNPs in a dose-dependent manner. The AM-CuNPs exhibited significantly good scavenging (58.25% ± 0.65) against H_2_O_2_ oxidative stress conditions in vitro. Furthermore, vitamin C, the standard antioxidant compound, showed significantly higher H_2_O_2_ radical scavenging activity. However, the AM-CuNPs can be used as a useful natural antioxidant for the treatment of various degenerative diseases caused by oxidative stress and associated problems. This study found good values for free radical scavenging when compared with the previously reported NPs [35].

### 3.7. In Silico Analysis of Anti-Inflammatory Properties of A. monanthum Extract and CuNPs

The in silico anti-inflammatory properties of *A. monanthum* extract, which is used for the biological synthesis of CuNPs, were studied through computational modeling dynamics and simulation analysis.

**Figure 5:**
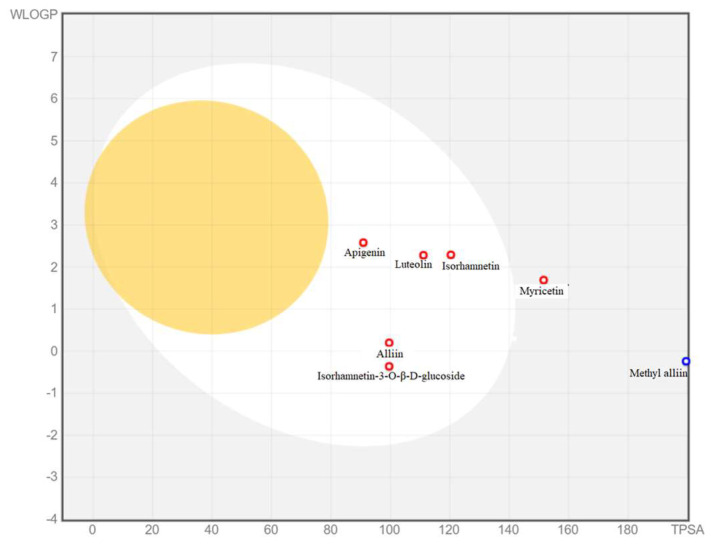
Boiled−egg graph of selected metabolites in AM plant extract. (White region of egg: high probability of passive absorption by the gastrointestinal tract, and high probability of brain penetration. Blue dots: Compounds that are substrates of P-glycoprotein, Red dots: Compounds that are not substrates of P-glycoprotein. Particularly, the yellow region of boiled-egg did not shows any selected metabolites present in the region. Therefore, the selected metabolites indicates potential biochemical functions).

ADME analysis: To gain insight into the underexplored potential of alliin, apigenin, isorhamnetin-3-O-β-D-glucoside, isorhamnetin, luteolin, methyl alliin and myricetin as candidates for anti-inflammatory agents, the study analyzed these compounds using the SwissADME web tool [36]. A pharmacokinetic analysis of the anti-inflammatory characteristics, including medicinal chemistry and drug-likeness score calculation criteria, was conducted using the SwissADME calculation procedure. Figure 5 presents the ADME score for selected ligands including alliin, apigenin, isorhamnetin-3-O-β-D-glucoside, isorhamnetin, luteolin and myricetin. The ADME results revealed that most of the seven compounds exhibited a unique pattern of high GI absorption and water solubility, and were not permeable through the blood–brain barrier (BBB) from the five Lipinski rule. The SwissADME graph of the compound probability of LOR5 and the calculation of the selected phyto-constituents are shown in Figure 5 and Table 3, respectively. Most of the selected compounds (alliin, apigenin, isorhamnetin, luteolin and methyl alliin) for evaluation of the anti-inflammatory effects exerted food ADME properties and had log kp values that are close to quercetin, which is found in standard antioxidant and anti-inflammatory natural compounds [37].

**Figure 6:**
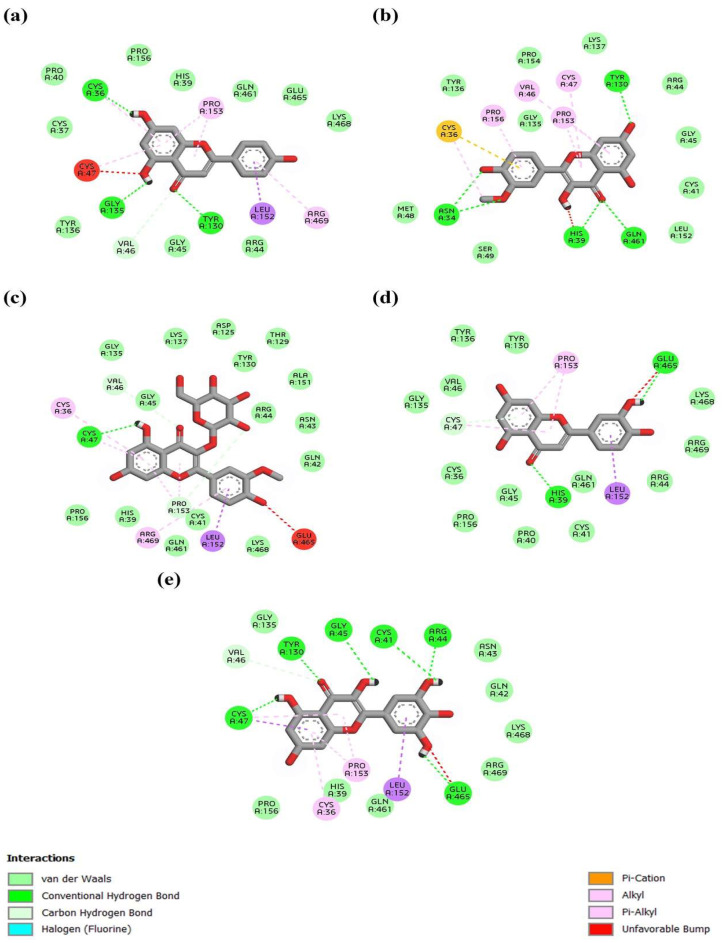
Protein COX-2 interaction with (**a**) Apigenin, (**b**) Isorhamnetin, (**c**) Isorhamnetin-glucoside, (**d**) Luteolin, and (**e**) Myricetin. Myricetin forming highest number of hydrogen bonds with protein among all compounds.

**Figure 7:**
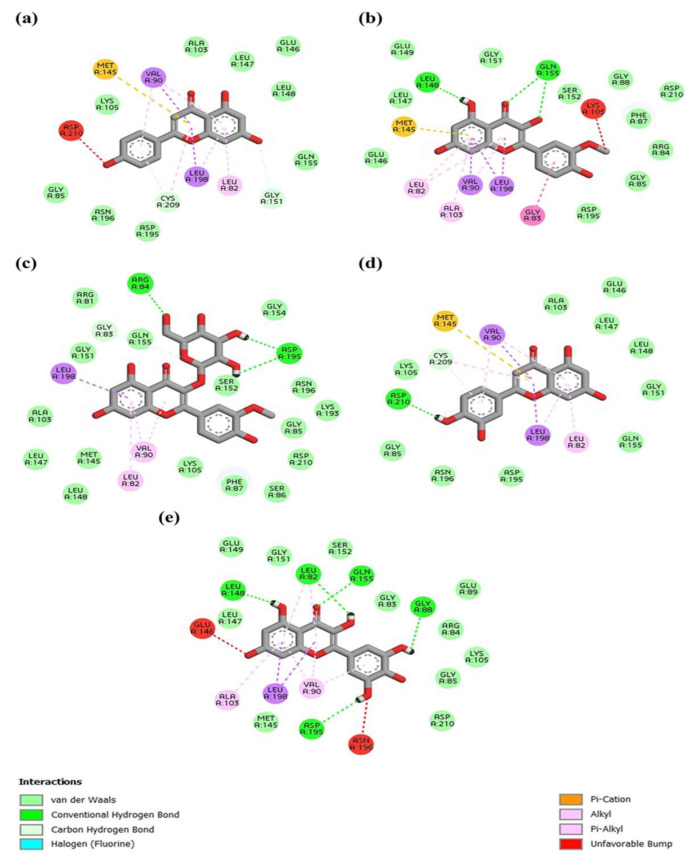
Protein NF-kB-inducing Kinase interaction with (**a**) Apigenin, (**b**) Isorhamnetin, (**c**) Isorhamnetin-glucoside, (**d**) Luteolin, and (**e**) Myricetin. Myricetin forming highest number of hydrogen bonds with protein among all compounds.

Figure 6 and Figure 7 show the three-dimensional crystal structures of the top anti-inflammatory ligands identified from *A. monanthum* that were docked on the active cavity site for the target COX-2 and NF-kB proteins. The atomic model of the compounds and ligands showed that various H bonds are present in the backbones of the chemical structures and Van der Waals interaction sites, and other polar entities are available in the backbone of the chemical 2D and 3D structures. The H bonds are considered to be the facilitators of the target–ligand interactions, as they displace protein bound water molecules (OH^−^, H^+^) and expedite the free energy barrier reduction during enzyme catalysis [38]. Based on these criteria, the molecular docking of five compounds: (a) apigenin, (b) isorhamnetin, (c) isorhamnetin-glucoside, (d) luteolin and (e) myricetin were used, since they had the best antioxidant and anti-inflammation activities. Table 4 shows the top binding affinity scores of the selected ligands for anti-inflammation function. The binding energies for all seven compounds were against NF-kB, and they had good binding affinity scores, including isorhamnetin-glucoside 5,318,645 (−9.1 kcal/mol), followed by isorhamnetin 5,281,654 (−8.9 kcal/mol), myricetin 5,281,672 (−8.9 kcal·mol^−1^), luteolin 5,280,445 (−9.3 kcal/mol) and apigenin 5,280,443 (−9.0 kcal/mol), which showed good results when compared with the standard compounds of quercetin. In addition, the COX-2 target had good binding affinity among all the ligands used in this study. Furthermore, the H bonding of the apigenin, luteolin and isorhamnetin-glucoside compounds interacted with the following amino acid residues: LYS34, ALA37, ALA38, GLY40, SER41, LEU80, ARG81, LEU82, GLY83, ARG84, GLY85, SER86, PHE87, ASP225, LEU226, THR228, GLY229, ASP230, ILE232, GLY234 and THR235, showing good interaction with a surface area of 787.625 Å2 and a surface volume of 619.234 Å3. Hydrophobic interactions are major criteria for protein–ligand complex formation and enhance the affinity and biological activities of the complex substances, which helps to stabilize their biological environment [39].

Molecular docking: AutoDock (version-1.5.7) [40] was used for constructing the grid box, including the binding site residues (as listed in Table 4) determined from the AutoDock tools [41]. Table 3 shows the grid box dimensions for the x, y and z coordinates for the four target proteins. The target proteins and the selected compounds were prepared for docking. Using the PubChem API, 3D-SDF structures of the compounds were downloaded for seven CIDs [42]. All seven 3D-SDF files were minimized and converted to PDBQT using Open Babel [43]. Hydrogen and Kollman charges were introduced into the target proteins, and water molecules and heteroatoms were removed and saved in PQBQT format, as part of the docking protocol. The AutoDock Vina software was used for the molecular docking of the seven selected compounds [44]. The docking parameters considered during virtual screening were binding modes 20, exhaustiveness 10 and a maximum energy difference of four (kcal/mol), for each compound. Docking then proceeded with the compounds. The compounds were ranked based on the binding scores and the most promising compounds were identified.

The COX-2 protein and the NF-kB-inducing kinase showed positive interactions with the bioactive compounds listed in Table 4 and Table 5. Five out of the seven bioactive compounds (isorhamnetin-glucoside, isorhamnetin, myricetin, luteolin, apigenin) showed less than −7 kcal/mol average binding energy, indicating high binding affinity with COX-2 and the NF-kB-inducing kinase. Protein–ligand interactions for the COX-2 protein and the NF-kB-inducing kinase were visualized with the five identified compounds and depicted using BIOVIA Discovery Studio (version 21.10) [BIOVIA, 2021]. As shown in Table 4 and Table 5, myricetin showed the highest number of hydrogen bonds with both the COX-2 protein and the NF-kB-inducing kinase among all compounds. All compounds showed significant binding affinity when docked with MAP3K14/NF-kB and COX-2. The three ligands including isorhamnetin-glucoside, luteolin and apigenin showed the top scores when docked with NF-kB and the COX-2 protein target for inflammation control [45]. For the COX-2 protein, different binding pocket amino acid residues were identified using the CASTp server (GLN374, ASN375, ARG376, ILE377, ALA378, PHE381, LEU384, TYR385, TRP387, LYS459, GLN461, GLU465, LYS468, ARG469, PHE470, MET471, LEU472, ARG513, ALA516, ILE517, PHE518, MET522, VAL523, GLU524, GLY526, ALA527, PHE529, SER530, LEU531, LYS532, GLY533 and LEU534), which showed interaction with the surface area at 1785.762 Å2 and the surface volume at 1369.03 Å3. Furthermore, the myricetin ligand showed the top binding energy when compared with the alliin and methyl alliin ligand molecules. In addition, luteolin 5280445, followed by isorhamnetin-glucoside 5318645, apigenin 5,280,443 and isorhamnetin 5281654, also exhibited the highest binding affinity score against the COX-2 protein (PBD: 5IKR). Generally, arginine, tryptophan, leucine and alanine were the key proteins required for an innate inflammatory response, while the reduction of tyrosine, histidine and glutamine signaling protein inactivation could result in the up-regulation of the autophagy-related downstream signaling pathways, which results in the suppression of inflammation progress [45]. Conversely, Ficus religiosa contains various compounds that target the COX-2 receptor in cancer and inflammation-related diseases. The compounds humulene, lanosterol, myricetin and z-3-hexenyl acetate exhibited lower binding energy compared with the reference ligand. Therefore, these compounds are not able to inhibit the COX-2 receptor-related activities [46]. Furthermore, the compounds from *A. monanthum* have excellent docking scores and could be potent molecules for further studies to evaluate as natural anti-inflammation drugs for humans.

## 4. Conclusions

In conclusion, AM-CuNPs were successfully synthesized using *A. monanthum* extract and plant phyto-constituents such as polyphenolics, terpenoids and flavonoids, which played a major role in the formation of the nanoparticles. UV-vis analysis exhibited a characteristic surface plasmon resonance peak at 390 nm, indicating the formation of CuNPs. The SEM and TEM electron microscope images provided strong evidence that the nanomorphology of AM-CuNPs consists of spherical and triangular shapes, with an average size of 30–80 nm. Powder XRD measurement revealed that the synthesized CuNPs are crystalline in nature and fcc CuNPs. FT-IR analysis confirmed that the CuNPs contain various functional groups such as carboxylic acids, amines, amides and esters that are derived from the *A. monanthum* extract and used as reducing agents. The biosynthesized CuNPs are stably displayed and have a wide zone of inhibition against pathogenic bacteria such as *E.coli* and *S. aureus* when compared with standard amoxicillin clavulanate. Therefore, the biosynthesized CuNPs could act as potential agents against multidrug-resistant microbes to enable their development as natural antibiotic compounds. In addition, the study evaluated the in silico anti-inflammatory properties of seven compounds derived from *A. monanthum*. The selected bioactive metabolites provided a potent anti-inflammatory effect and showed the lowest binding energy to the inflammatory protein receptor NF-kB. Hence, further experimental analysis is needed using more ligands from *A. monanthum* to generate an improved confirmation of the ligand–receptor binding complex.

## Figures and Tables

**Table 1:** Antibacterial activity of AM-CuNPs. Different alphabetical letters ^a, b, c^ and ^d^ superscript indicate the significant differences at *p* > 0.05 in each group.

Microorganisms	Zone of Inhibition (mm)		
	CuSO_4_	AM-CuNPs	Standard
1. *E. coli*	3.2 ± 0.2 ^d^	13.58 ± 0.35 ^c^	18.27 ± 0.21 ^b^
2. *S. aureus*	1.8 ± 0.18 ^a^	11.63 ± 0.42 ^a^	19.54 ± 0.18 ^c^
3. *P. aureginosa*	2.4 ± 0.22 ^c^	12.77 ± 0.34 ^b^	19.53 ± 0.16 ^c^
4. *B. subtilis*	2.6 ± 0.11 ^c^	14.25 ± 0.29 ^d^	17.45 ± 0.18 ^a^
5. *S. typhi*	2.1 ± 0.22 ^b^	12.28 ± 0.33 ^a^	18.56 ± 0.2 ^b^

**Table 2:** Anti-oxidant activity of AM-CuNPs. Different alphabetical letters ^a^, ^b^, ^c^ and ^d^ superscript indicate the significant differences at *p* > 0.05 in each group.

Sample Name	Anti-Oxidant Assay		
	DPPH (%)	ABTS (%)	H_2_O_2_ (%)
AM-CuNPs	68.46 ± 0.85 ^c^	62.55 ± 0.68 ^c^	66.17 ± 0.73 ^c^
CuSO4 1 mM	22.35 ± 0.58 ^a^	19.26 ± 0.75 ^a^	17.58 ± 0.66 ^a^
*A. monanthum* extract	63.28 ± 0.63 ^b^	61.54 ± 0.82 ^b^	65.29 ± 0.68 ^b^
Vitamin C-standard	92.56 ± 0.45 ^d^	83.66 ± 0.38 ^d^	85.23 ± 0.35 ^d^

**Table 3:** The SwissADME calculations of bioactive compounds.

Molecule	MW (g/mol)	Water Solubility	GI Absorption	BBB Perm-Eant	P-glyco -Protein Substrate	Lipinski
Alliin	177.22	Highly soluble	High	No	No	Yes
Apigenin	270.24	Moderately Soluble	High	No	No	Yes
Isorhamnetin-3-O-β-D-glucoside	478.4	Soluble	Low	No	Yes	No
Isorhamnetin	316.26	Soluble	High	No	No	Yes
Luteolin	286.24	Soluble	High	No	No	Yes
Methyl-alliin	151.18	Highly soluble	High	No	No	Yes
Myricetin	318.24	Soluble	Low	No	No	Yes

**Table 4:** Binding energy for the seven compounds against COX-2 or PTGS2 (PBD: 5IKR).

Ligand	Average Binding Energy	Top Binding Energy
Myricetin_5281672	−8.17	−9.8
Luteolin_5280445	−8	−9.4
Isorhamnetin-glucoside_5318645	−7.9	−9.7
Apigenin_5280443	−7.58	−9
Isorhamnetin_5281654	−7.22	−8.9
Alliin_87310	−4.78	−5.2
Methyl_alliin_99483	−4.43	−4.8

**Table 5:** Binding energy for the seven compounds against MAP3K14 or NIK (PDB: 4DN5); NF-kB-inducing Kinase.

Ligand	Average Binding Energy	Top Binding Energy
Isorhamnetin-glucoside_5318645	−7.78	−9.1
Isorhamnetin_5281654	−7.6	−8.9
Myricetin_5281672	−7.5	−8.9
Luteolin_5280445	−7.47	−9.3
Apigenin_5280443	−7.46	−9
Alliin_87310	−4	−4.6
Methyl_alliin_99483	−3.52	−4.3

## Data Availability

The reported results can be found in the articles.

## References

[1] Ali M.A., Ahmed T., Wu W., Hossain A., Hafeez R., Masum M.M.I., Wang Y., An Q., Sun G., Li B. (2020). Advancements in Plant and Microbe-Based Synthesis of Metallic Nanoparticles and Their Antimicrobial Activity against Plant Pathogens. Nanomaterials.

[2] Das R.K., Pachapur V.L., Lonappan L., Naghdi M., Pulicharla R., Maiti S., Cledon M., Dalila L.M.A., Sarma S.J., Brar S.K. (2017). Biological Synthesis of Metallic Nanoparticles: Plants, Animals and Microbial Aspects. Nanotechnol. Environ. Eng..

[3] Ghosh M.K., Sahu S., Gupta I., Ghorai T.K. (2020). Green Synthesis of Copper Nanoparticles from an Extract of *Jatropha curcasleaves*: Characterization, Optical Properties, CT-DNA Binding and Photocatalytic Activity. RSC Adv..

[4] Lee H.J., Song J.Y., Kim B.S. (2013). Biological Synthesis of Copper Nanoparticles Using *Magnolia kobus* Leaf Extract and Their Antibacterial Activity. J. Chem. Technol. Biotechnol..

[5] Abboud Y., Saffaj T., Chagraoui A., El Bouari A., Brouzi K., Tanane O., Ihssane B. (2014). Biosynthesis, Characterization and Antimicrobial Activity of Copper Oxide Nanoparticles (CONPs) Produced Using Brown Alga Extract (*Bifurcaria bifurcata*). Appl. Nanosci..

[6] Sivaraj R., Rahman P.K.S.M., Rajiv P., Salam H.A., Venckatesh R. (2014). Biogenic Copper Oxide Nanoparticles Synthesis Using *Tabernaemontana divaricate* Leaf Extract and Its Antibacterial Activity against Urinary Tract Pathogen. Spectrochim. Acta—Part A Mol. Biomol. Spectrosc..

[7] Ramyadevi J., Jeyasubramanian K., Marikani A., Rajakumar G., Rahuman A.A., Santhoshkumar T., Kirthi A.V., Jayaseelan C., Marimuthu S. (2011). Copper Nanoparticles Synthesized by Polyol Process Used to Control Hematophagous Parasites. Parasitol. Res..

[8] Rajasekar A., Rajeshkumar S. (2021). Antioxidant and Anti-Inflammatory Property of Copper Nanoparticles (Cunps) Synthesised Using Blue Tea. J. Complement. Med. Res..

[9] Yaqoob A.A., Ahmad H., Parveen T., Ahmad A., Oves M., Ismail I.M.I., Qari H.A., Umar K., Mohamad Ibrahim M.N. (2020). Recent Advances in Metal Decorated Nanomaterials and Their Various Biological Applications: A Review. Front. Chem..

[10] Alshammari S.O., Mahmoud S.Y., Farrag E.S. (2023). Synthesis of Green Copper Nanoparticles Using Medicinal Plant *Krameria* sp. Root Extract and Its Applications. Molecules.

[11] Ermini M.L., Voliani V. (2021). Antimicrobial Nano-Agents: The Copper Age. ACS Nano.

[12] Seyyed Hajizadeh Y., Babapour E., Harzandi N., Yazdanian M., Ranjbar R. (2023). The Effect of Cytotoxicity and Antimicrobial of Synthesized CuO NPs from Propolis on HEK-293 Cells and *Lactobacillus acidophilus*. Evid.-Based Complement. Altern. Med..

[13] Huq M.A., Ashrafudoulla M., Rahman M.M., Balusamy S.R., Akter S. (2022). Green Synthesis and Potential Antibacterial Applications of Bioactive Silver Nanoparticles: A Review. Polymers.

[14] Singh H., Desimone M.F., Pandya S., Jasani S., George N., Adnan M., Aldarhami A., Bazaid A.S., Alderhami S.A. (2023). Revisiting the Green Synthesis of Nanoparticles: Uncovering Influences of Plant Extracts as Reducing Agents for Enhanced Synthesis Efficiency and Its Biomedical Applications. Int. J. Nanomed..

[15] Shenhar B.R., Norsten T.B., Rotello V.M. (2005). Polymer-Mediated Nanoparticle Assembly: Structural Control and Applications. Adv. Mater..

[16] Moon H.I. (2011). Larvicidal Activity of Major Essential Oils from Stems of *Allium monanthum* Maxim. against *Aedes aegypti* L. J. Enzyme Inhib. Med. Chem..

[17] Yi G., Kim K.-M. (2015). Comparison of Major Agricultural Traits and Genetic Diversity in Indigenous Korean Rocambole (*Allium monanthum* Max.) Based on Collecting Sites. J. Korean Soc. Int. Agric..

[18] Chung H.S. (2022). Antiproliferative Activity of *Allium monanthum* in HT-29 Human Colorectal Adenocarcinoma Cells. Korean Tea Soc..

[19] Yoon K.R., Ryu J.K., Lee E. (2013). Biological Effects of *Allium monanthum* Extracts on Lipid Metabolism, Anti-Oxidation and the Production of Pro-Inflammatory Cytokines in Rats Fed a High-Fat Diet. Korean J. Plant Resour..

[20] Maham M., Sajadi S.M., Kharimkhani M.M., Nasrollahzadeh M. (2017). Biosynthesis of the CuO Nanoparticles Using *Euphorbia chamaesyce* Leaf Extract and Investigation of Their Catalytic Activity for the Reduction of 4-Nitrophenol. IET Nanobiotechnol..

[21] Bogdanović U., Lazić V., Vodnik V., Budimir M., Marković Z., Dimitrijević S. (2014). Copper Nanoparticles with High Antimicrobial Activity. Mater. Lett..

[22] Rajeshkumar S., Menon S., Venkat Kumar S., Ponnanikajamideen M., Ali D., Arunachalam K. (2021). Anti-Inflammatory and Antimicrobial Potential of Cissus Quadrangularis-Assisted Copper Oxide Nanoparticles. J. Nanomater..

[23] Bojarska J., Remko M., Breza M., Madura I.D., Kaczmarek K., Zabrocki J., Wolf W.M. (2020). A Supramolecular Approach to Structure-Based Design with a Focus on Synthons Hierarchy in Ornithine-Derived Ligands: Review, Synthesis, Experimental and in Silico Studies. Molecules.

[24] Fatoki T.H., Ajiboye B.O., Aremu A.O. (2023). In Silico Evaluation of the Antioxidant, Anti-Inflammatory, and Dermatocosmetic Activities of Phytoconstituents in Licorice (*Glycyrrhiza glabra* L.). Cosmetics.

[25] Khairy A., Ghareeb D.A., Celik I., Hammoda H.M., Zaatout H.H., Ibrahim R.S. (2023). Forecasting of Potential Anti-Inflammatory Targets of Some Immunomodulatory Plants and Their Constituents Using in Vitro, Molecular Docking and Network Pharmacology-Based Analysis. Sci. Rep..

[26] Mali S.C., Dhaka A., Githala C.K., Trivedi R. (2020). Green Synthesis of Copper Nanoparticles Using *Celastrus paniculatus* Willd. Leaf Extract and Their Photocatalytic and Antifungal Properties. Biotechnol. Rep..

[27] Amaliyah S., Pangesti D.P., Masruri M., Sabarudin A., Sumitro S.B. (2020). Green Synthesis and Characterization of Copper Nanoparticles Using *Piper retrofractum Vahl* Extract as Bioreductor and Capping Agent. Heliyon.

[28] Khashan K.S., Jabir M.S., Abdulameer F.A. (2018). Preparation and Characterization of Copper Oxide Nanoparticles Decorated Carbon Nanoparticles Using Laser Ablation in Liquid. J. Phys. Conf. Ser..

[29] Sankara Narayanan V.P., Kathirason S.G., Elango P., Subramanian R., Sivagurusundar R., Gurusamy A. (2023). *Emilia sonchifolia* Leaf Extract-Mediated Green Synthesis, Characterization, in Vitro Biological Activities, Photocatalytic Degradation and in Vivo Danio Rerio Embryo Toxicity of Copper Nanoparticles. RSC Adv..

[30] Sadia B.O., Cherutoi J.K., Achisa C.M. (2021). Optimization, Characterization, and Antibacterial Activity of Copper Nanoparticles Synthesized Using *Senna didymobotrya* Root Extract. J. Nanotechnol..

[31] Vijayakumar G., Kesavan H., Kannan A., Arulanandam D., Kim J.H., Kim K.J., Song H.J., Kim H.J., Rangarajulu S.K. (2021). Phytosynthesis of Copper Nanoparticles Using Extracts of Spices and Their Antibacterial Properties. Processes.

[32] Murthy H.C.A., Desalegn T., Kassa M., Abebe B., Assefa T. (2020). Synthesis of Green Copper Nanoparticles Using Medicinal Plant *Hagenia abyssinica* (Brace) JF. Gmel. Leaf Extract: Antimicrobial Properties. J. Nanomater..

[33] Ramasubbu K., Padmanabhan S., Al-Ghanim K.A., Nicoletti M., Govindarajan M., Sachivkina N., Rajeswari V.D. (2023). Green Synthesis of Copper Oxide Nanoparticles Using *Sesbania grandiflora* Leaf Extract and Their Evaluation of Anti-Diabetic, Cytotoxic, Anti-Microbial, and Anti-Inflammatory Properties in an In-Vitro Approach. Fermentation.

[34] Apak R., Özyürek M., Güçlü K., Çapanoğlu E. (2016). Antioxidant Activity/Capacity Measurement. 1. Classification, Physicochemical Principles, Mechanisms, and Electron Transfer (ET)-Based Assays. J. Agric. Food Chem..

[35] Rehana D., Mahendiran D., Kumar R.S., Rahiman A.K. (2017). Evaluation of Antioxidant and Anticancer Activity of Copper Oxide Nanoparticles Synthesized Using Medicinally Important Plant Extracts. Biomed. Pharmacother..

[36] Daina A., Michielin O., Zoete V. (2017). SwissADME: A Free Web Tool to Evaluate Pharmacokinetics, Drug-Likeness and Medicinal Chemistry Friendliness of Small Molecules. Sci. Rep..

[37] Daina A., Zoete V. (2016). A BOILED-Egg to Predict Gastrointestinal Absorption and Brain Penetration of Small Molecules. ChemMedChem.

[38] Sultana M.J., Nibir A.I.S., Ahmed F.R.S. (2023). Biosensing and Anti-Inflammatory Effects of Silver, Copper and Iron Nanoparticles from the Leaf Extract of *Catharanthus roseus*. Beni-Suef Univ. J. Basic Appl. Sci..

[39] Du X., Li Y., Xia Y.L., Ai S.M., Liang J., Sang P., Ji X.L., Liu S.Q. (2016). Insights into Protein–Ligand Interactions: Mechanisms, Models, and Methods. Int. J. Mol. Sci..

[40] Morris G.M., Ruth H., Lindstrom W., Sanner M.F., Belew R.K., Goodsell D.S., Olson A.J. (2009). Software News and Updates AutoDock4 and AutoDockTools4: Automated Docking with Selective Receptor Flexibility. J. Comput. Chem..

[41] Tian W., Chen C., Lei X., Zhao J., Liang J. (2018). CASTp 3.0: Computed Atlas of Surface Topography of Proteins. Nucleic Acids Res..

[42] Kim S., Chen J., Cheng T., Gindulyte A., He J., He S., Li Q., Shoemaker B.A., Thiessen P.A., Yu B. (2023). PubChem 2023 Update. Nucleic Acids Res..

[43] O’Boyle N.M., Banck M., James C.A., Morley C., Vandermeersch T., Hutchison G.R. (2011). Open Babel: An Open Chemical Toolbox. J. Cheminform..

[44] Eberhardt J., Santos-Martins D., Tillack A.F., Forli S. (2021). AutoDock Vina 1.2.0: New Docking Methods, Expanded Force Field, and Python Bindings. J. Chem. Inf. Model..

[45] Gonzales A.L., Huang S.K.H., Sevilla U.T.A., Hsieh C.Y., Tsai P.W. (2023). In Silico Analysis of Anti-Inflammatory and Antioxidant Properties of Bioactive Compounds from *Crescentia cujete* L. Molecules.

[46] Utami W., Aziz H.A., Fitriani I.N., Zikri A.T., Mayasri A., Nasrudin D. (2020). In Silico Anti-Inflammatory Activity Evaluation of Some Bioactive Compound from *Ficus religiosa* through Molecular Docking Approach. J. Phys. Conf. Ser..

